# Beneficial Effects of SREBP Decoy Oligodeoxynucleotide in an Animal Model of Hyperlipidemia

**DOI:** 10.3390/ijms21020552

**Published:** 2020-01-15

**Authors:** Hyun-Jin An, Jung-Yeon Kim, Mi-Gyeong Gwon, Hyemin Gu, Hyun-Ju Kim, Jaechan Leem, Sung Won Youn, Kwan-Kyu Park

**Affiliations:** 1 Department of Pathology, College of Medicine, Catholic University of Daegu, Daegu 42472, Korea; 2 Department of Immunology, College of Medicine, Catholic University of Daegu, Daegu 42472, Korea; 3 Department of Radiology, College of Medicine, Catholic University of Daegu, Daegu 42472, Korea

**Keywords:** keyword 1, SREBP 2, Hyperlipidemia 3, Decoy oligodeoxynucleotide

## Abstract

Hyperlipidemia is a chronic disorder that plays an important role in the development of cardiovascular diseases, type II diabetes, atherosclerosis, hypertension, and non-alcoholic fatty liver disease. Hyperlipidemias have created a worldwide health crisis and impose a substantial burden not only on personal health but also on societies and economies. Transcription factors in the sterol regulatory element binding protein (SREBP) family are key regulators of the lipogenic genes in the liver. SREBPs regulate lipid homeostasis by controlling the expression of a range of enzymes required for the synthesis of endogenous cholesterol, fatty acids, triacylglycerol, and phospholipids. Thereby, SREBPs have been considered as targets for the treatment of metabolic diseases. The aim of this study was to investigate the beneficial functions and the possible underlying molecular mechanisms of SREBP decoy ODN, which is a novel inhibitor of SREBPs, in high-fat diet (HFD)-fed hyperlipidemic mice. Our studies using HFD-induced hyperlipidemia animal model revealed that SREBB decoy ODN inhibited the increased expression of fatty acid synthetic pathway, such as SREBP-1c, FAS, SCD-1, ACC1, and HMGCR. In addition, SREBP decoy ODN decreased pro-inflammatory cytokines, including TNF-α, IL-1β, IL-8, and IL-6 expression. These results suggest that SREBP decoy ODN exerts its anti-hyperlipidemia effects in HFD-induced hyperlipidemia mice by regulating their lipid metabolism and inhibiting lipogenesis through inactivation of the SREPB pathway.

## 1. Introduction

Non-alcoholic fatty liver disease (NAFLD) is a medical condition characterized by the deposition of fat in the liver [1]. NAFLD is used to describe a range of conditions from a simple deposition of triglycerides in hepatocytes to a condition involving hepatitis, fibrosis, and cirrhosis [2]. This pathogenesis of NAFLD is complex and strongly associated to be a hepatic expression of the metabolic syndrome, arising together with type II diabetes, insulin resistance, and obesity [3]. Hyperlipidemia is a chronic disorder that plays a major role in the progression of cardiovascular diseases, type II diabetes, hypertension, atherosclerosis, and NAFLD [4]. Hyperlipidemia is generally caused by overabundance nutrients, which are characterized by high levels of triglyceride, total cholesterol, low-density lipoprotein cholesterol, and a diminished level of high-density lipoprotein cholesterol [5]. Excessive absorption of lipids from the diet is regarded a principal risk factor for hyperlipidemia [6,7,8]. Besides, various drugs, hormones, and multiple genetic defects affecting energy metabolism can lead to fat deposition in the liver [1]. Hyperlipidemias have produced a worldwide health critical situation and create a substantial burden not only on personal health but also on societies and economies [9]. Therefore, beneficial and secure pharmacological interventions for hyperlipidemia are urgently needed.

Lipids, acquired from the diet or synthesized endogenously in the liver and stored in the adipose tissue, are modulated by a delicate balance between lipid deposition and energy employ [10]. Sterol regulatory element binding proteins (SREBPs) are a family of transcription factors that regulate lipid homeostasis by modulating the expression of a range of enzymes required for the synthesis of intracellular levels cholesterol, fatty acids, triacylglycerol and phospholipids [11]. Furthermore, genes that encode the enzymes required for the both synthesis of cholesterol and triglyceride are controlled by membrane-bound transcription factors SREBPs [12,13]. The SREBP have three major isoforms, SREBP-1a, SREBP-1c, and SREBP-2, play different roles in lipid synthesis [11]. SREBP-1a operates all SREBP-responsive genes [13]. SREBP-1c is involved in fatty acid and triglyceride synthesis, whereas SREBP-2 is more specific to cholesterol synthesis [14,15,16,17]. SREBP-1c and SREBP-2 are the predominant isoforms in the liver and most other intact tissues [13]. SREBP-1c, regulated by insulin, is a marker of disrupted lipid homeostasis, which is plentifully expressed in the liver, and it stimulates the genes that regulate fatty acid synthesis [18,19]. For these reasons, the regulation of SREBP-1c has been preponderantly studied. Therefore, the inhibition of the SREBPs by these small molecules may be an effective strategy for treating obesity, atherosclerosis, hypertension, and fatty liver disease.

The treatment of hyperlipidemia includes lifestyle adjustments (the integration of diet and exercise), if necessary, in combination with drug therapy, such as statins, niacin, fibrates, and omega-3 fatty acids [20]. Surgery is also a powerful approach [21]. However, most of these approaches have adverse side effects, including myalgia, fatigue, dyspnea, memory loss, and peripheral neuropathy [22]. We therefore attempted to suppress hyperlipidemia by regulating lipid synthesis and homeostasis at the transcription level by blocking the transcription factor SREBPs. Decoy technology uses synthetic double-stranded oligodeoxynucleotide (ODN) including the consensus binding site sequence of a targeted transcription factor. The decoy ODN obstructs the activity of a specific transcription factor and prevents specific gene expression at the DNA level [23]. Consequentially, the decoy ODN binds to the specific transcription factor and restrains gene expression by preventing the activation of involved genes. This decoy ODN strategy is an effective approach for suppressing specific gene expression both in vitro and in vivo [24,25]. We previously demonstrated that the antifibrotic effect of the NF-κB decoy ODN in a hepatic fibrosis animal model [26]. Additionally, we demonstrated the effect of Smad ODN on hepatic fibrosis in hepatocytes and CCl_4_-induced hepatic fibrosis [27]. FITC-labeled Smad ODNs, which were detected by fluorescence, were shown in the cytoplasm and nucleus of liver cells. These results indicate that Smad decoy ODN was successfully transfected into a mouse liver. However, the effect of SREBP decoy ODN on hyperlipidemia on animal model has not been reported.

The aim of this study was to investigate the beneficial functions and the possible underlying molecular mechanisms of SREBP decoy ODN in high-fat diet (HFD)-fed hyperlipidemic mice. Our results indicated that SREBP decoy ODN reversed the metabolic abnormalities in these mice by regulating the SREBP signaling pathway, and could therefore potentially serve as an additional therapeutic intervention against hyperlipidemia.

## 2. Results

### 2.1. Construction of Decoy ODNs

**Figure 1:**
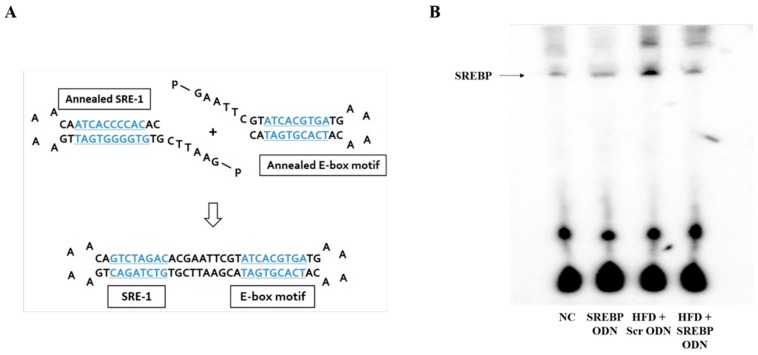
Construction of sterol regulatory element binding protein (SREBP) decoy ODN. (**A**) The target sites for SREBP were selected via the sequential overlap-simulation of secondary structures using the S-Fold program. SREBP ODN ligates the annealed SRE-1 and annealed E-box motif (consensus sequence is blue and underlined). (**B**) Electrophoretic mobility shift assay (EMSA) was conducted to investigate the effect of SREBP decoy ODN on SREBP expression in high-fat diet (HFD)-induced hyperlipidemic mice. NC, normal control group; SREBP ODN, group treated with SREBP decoy ODN; HFD + Scr ODN, HFD fed group treated with scrambled decoy ODN; HFD + SREBP ODN, HFD fed group treated with SREBP decoy ODN.

We first designed the SREBP decoy ODN. The target sites for SREBP were selected via the sequential overlap-simulation of secondary structures using the S-Fold program (Figure 1A). The mature SREBP-1 is translocated into the nucleus where it can bind to the sterol regulated element-1 (SRE-1) (5’-ATCACCCCAC-3’) sequence present in the promoters/enhancers of lipogenic genes (including itself), activating their transcription (13-1). SREBP decoy ODN has dual sequence specificity binding to both an E-box motif (5’-ATCACGTGA-3’) and to SRE-1. To identify the function of SREBP decoy ODN on the control of SREBP expression, electrophoretic mobility shift assay (EMSA) was executed. (Figure 1B). EMSA was conducted with nuclear extracts of HFD-induced hyperlipidemia mice liver tissues that had been transfected with SREBP decoy ODN to establish the efficacy of SREBP ODN on the HFD-induced DNA-binding activity of SREBP. HFD + Scr ODN mice increased of SREBP DNA-binding activity. Whereas, HFD + SREBP ODN treatment group reduced the SREBP DNA-binding activity compared with Scr ODN group. These results demonstrated that transfected SREBP decoy ODN effectually diminish the expression of SREBP.

### 2.2. SREBP Decoy ODN Attenuated Morphological Change on Hyperlipidemic Mice Liver

**Figure 2:**
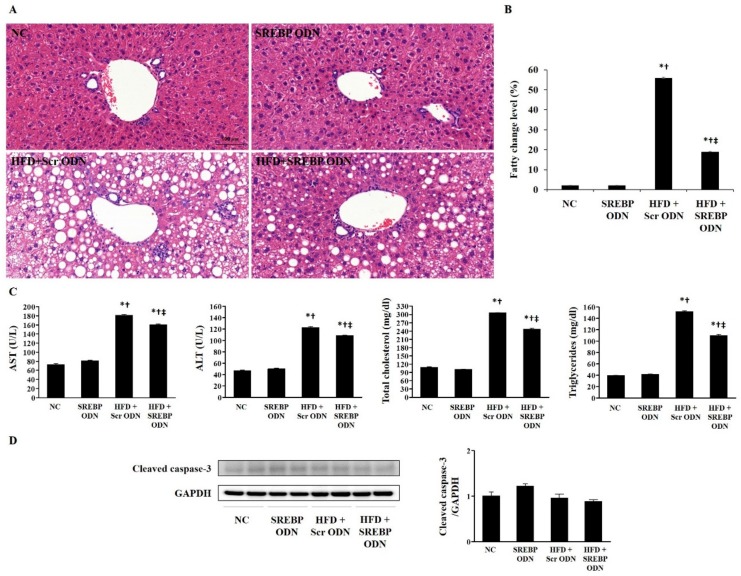
Histological change and serum biochemistry of HFD-fed mice. SREBP decoy ODN treatment effectively suppresses fatty change, serum aspartate aminotransferase (AST), alanine transaminase (ALT), total cholesterol, and triglycerides (*n* = 6). (**A**) H&E staining; Scale bar = 100 μm. (**B**) Fatty change levels; (**C**) Serum AST, ALT, total cholesterol, and triglycerides. Representative images from each group. (**D**) Western blot results show that cleaved caspase-3 expression in liver tissue. The expression levels of the protein from quantification of these images by Image J. NC, normal control group; SREBP ODN, group treated with SREBP decoy ODN; HFD + Scr ODN, HFD fed group treated with scrambled decoy ODN; HFD + SREBP ODN, HFD fed group treated with SREBP decoy ODN. * *p* < 0.05 compared to the NC group. † *p* < 0.05 compared to the SREBP ODN group. ‡ *p* < 0.05 compared to the HFD + Scr ODN group.

We investigated the effects of SREBP decoy ODN on hyperlipidemic mice at the histological and serum levels. Histological analysis showed that the HFD + SREBP decoy ODN treated groups had smaller histological and fatty change than the HFD + Scr decoy ODN treated group (Figure 2A,B). As shown in Figure 2C, the serum levels of alanine aminotransferase (ALT), aspartate aminotransferase (AST), which are associated with liver injury, were obviously elevated in HFD mice, whereas they were significantly decreased in the HFD + SREBP decoy ODN treated group. Hepatic total cholesterol and triglycerides levels were significantly higher in the HFD + Scr decoy ODN group than in the NC group. These expressions were significantly diminished in the HFD + SREBP decoy ODN mice. In this study, the cleaved caspase-3, a marker of apoptosis, expressions between NC group and HFD group was not significant (Figure 2D). Thus, SREBP decoy ODN effect is not related to apoptosis in hyperlipidemia mice liver. These results suggest that SREBP decoy ODN effectively inhibits lipid accumulation in the liver during HFD.

### 2.3. Effects of SREBP Decoy ODN on the Inflammation-Related Factors in Hyperlipidemic Mice Livers

**Figure 3:**
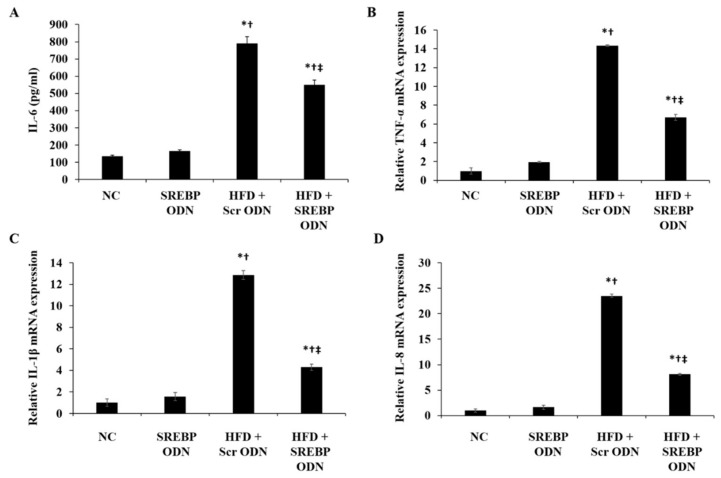
SREBP decoy ODN significantly inhibits the pro-inflammatory cytokines in the HFD-induced hyperlipidemic mouse model (*n* = 6). (**A**) ELISA results demonstrate that SREBP decoy ODN inhibits the IL-6 expression in hyperlipidemic mice. (**B**–**D**) Real-time PCR results show that SREBP decoy ODN inhibits expressions of TNF-α, IL-1β, and IL-8. NC, normal control group; SREBP ODN, group treated with SREBP decoy ODN; HFD + Scr ODN, HFD fed group treated with scrambled decoy ODN; HFD + SREBP ODN, HFD fed group treated with SREBP decoy ODN. * *p* < 0.05 compared to the NC group. † *p* < 0.05 compared to the SREBP ODN group. ‡ *p* < 0.05 compared to the HFD + Scr ODN group.

To investigate the effect of SREBP decoy ODN on the inflammatory cytokines, which plays a key role in lipid metabolism, we examined this by using ELISA and real-time PCR. HFD administration increased the serum concentration of IL-6 in HFD + Scr ODN mice more than in NC mice (Figure 3A). On the other hand, SREBP decoy ODN treatment significantly inhibited the secretion of IL-6 in HFD + SREBP ODN treatment group. In addition, HFD-induced hyperlipidemia mice liver tissues showed an increased mRNA level expression of TNF-α, IL-1β, and IL-8 (Figure 3B–D). Treatment with SREBP decoy ODN remarkably decreased the expression of TNF-α, IL-1β, and IL-8, more than the Scr decoy ODN did in HFD-induced hyperlipidemia mice. These results indicate that SREBP decoy ODN effectively improves HFD-induced hyperlipidemia, inflammation, and hepatic steatosis.

### 2.4. Effects of SREBP Decoy ODN on the Expression of Cholesterol Metabolism-Related Factors and Lipid Metabolism-Related Factors in the Liver

**Figure 4:**
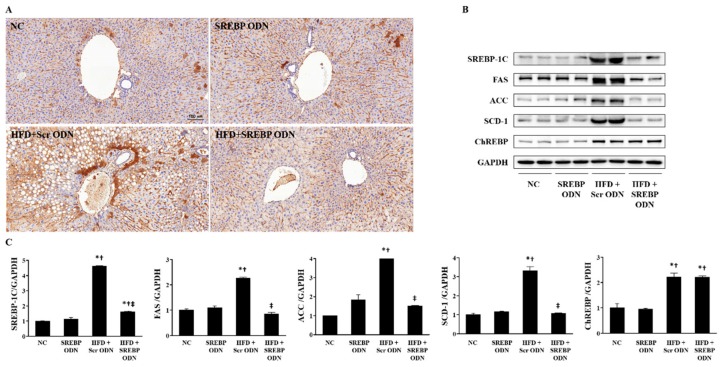
SREBP decoy ODN significantly inhibits cholesterol metabolism and fatty acid metabolism in the HFD-induced hyperlipidemic mouse model (*n* = 6). (**A**) Immunohistochemical staining results show that SREBP decoy ODN inhibits expression of HMGCR. (**B**) Western blot results show that SREBP decoy ODN inhibits expression of SREBP-1c, FAS, ACC, SCD-1. (**C**) The expression levels of the protein from quantification of these images by Image J. Representative images from each group. Scale bar = 100 μm. NC, normal control group; SREBP ODN, group treated with SREBP decoy ODN; HFD + Scr ODN, HFD fed group treated with scrambled decoy ODN; HFD + SREBP ODN, HFD fed group treated with SREBP decoy ODN. * *p* < 0.05 compared to the NC group. † *p* < 0.05 compared to the SREBP ODN group. ‡ *p* < 0.05 compared to the HFD + Scr ODN group.

HMG-CoA reductase (HMGCR) is the rate-limiting enzyme in cholesterol biosynthesis, so its activity is instrumental in controlling de novo cholesterol synthesis [28]. To understand the mechanism by which SREBP decoy ODN regulates liver lipid metabolism, the expression level of HMGCR in liver tissue was measured by IHC (Figure 4A). The expression of HMGCR, the metabolism-related genes, in the HFD + Scr ODN group was significantly higher than that in the NC group. However, SREBP decoy ODN suppressed this increase in the tissue expression of HMGCR. To investigate the action mechanism of SREBP decoy ODN, the protein expression levels of the lipogenic transcription factor (SREBP-1c) and of the lipogenesis-related genes, such as fatty acid synthase (FAS), acetyl-CoA carboxylase (ACC), stearoyl-CoA desaturase (SCD)-1, and carbohydrate response element binding protein (ChREBP) were measured in hyperlipidemic liver tissue by western blot analysis (Figure 4B,C). Interestingly, the expression levels of SREBP-1c, FAS, ACC, and SCD-1 in the HFD + Scr ODN group were significantly higher than those in the NC and SREBP ODN group. However, the HFD + SREBP decoy ODN treatment group restituted the increase in all these protein expressions to a near-normal control level. ChREBP, which is another lipogenic transcription factor, causes lipogenic gene expression primarily in reply to glucose and could potentially conduce to the insulin-independent modulation of the lipogenic genes [29,30]. ChREBP contain of two 5′-CACGTG type E box motifs separated by 5pb [31]. HFD + Scr ODN group showed an increased ChREBP protein level expression. Though, SREBP decoy ODN group did not show the ChREBP expression changes in hyperlipidemia mice. These results suggest that SREBP decoy ODN ameliorates dyslipidemia by inhibiting the activation of the SREBP signaling pathway.

### 2.5. Effects of SREBP Decoy ODN in Hyperlipidemic Mice Aorta

**Figure 5:**
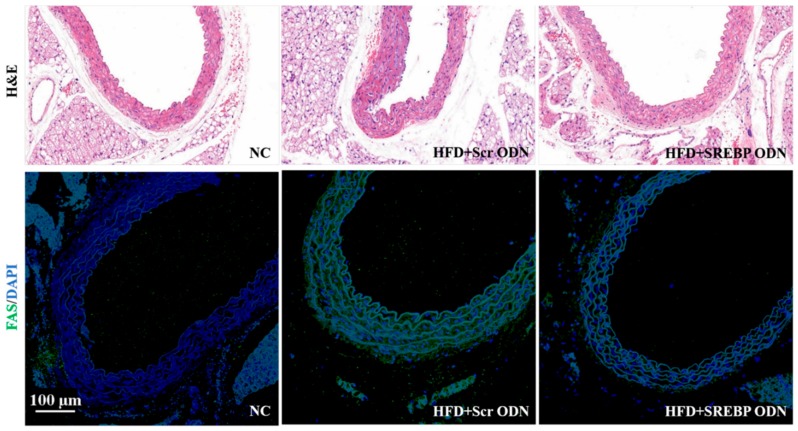
SREBP decoy ODN suppresses HFD-induced aorta damage. Histological section of murine aortas stained with H&E (**upper panel**) and immunofluorescence staining (**lower panel**) (*n* = 6). H&E staining show that changes were attenuated in the SREBP decoy treated group. Immunofluorescence staining results show that SREBP decoy ODN inhibits expression of FAS. Representative images from each group. Scale bar = 100 µm. NC, normal control group; HFD + Scr ODN, HFD fed group treated with scramble decoy ODN; HFD + SREBP ODN, HFD fed group treated with SREBP decoy ODN.

To understand the effects of SREBP decoy ODN further, we examined the effect of SREBP decoy ODN on the aortas of the hyperlipidemic mice. Pathological evaluations of aortic lesions were carried out. Consecutive cross-sections of cuffed descending aortas stained with H&E and immunofluorescence revealed the induction of hyperlipidemic lesion formation (Figure 5). The HFD + Scr ODN group showed a larger number of hyperlipidemic lesions in the aorta compared to the NC group. In the HFD + Scr ODN group, nearly all the animals developed fatty streaks in their aorta with an accumulation of lipids localized mainly in the areas subjacent to the endothelium. However, SREBP decoy ODN treatment changed the size of the hyperlipidemic lesions in the aortas, suggesting that SREBP decoy ODN exerted an apparently protective hyperlipidemic action. Furthermore, immunofluorescence staining results show that SREBP decoy ODN inhibits the expression of FAS in the aortas of HFD-induced hyperlipidemic mice. These results were consistent with the present study on mice livers.

## 3. Discussion

In this study, we investigated the inhibition of SREBP activity by the SREBP decoy ODN in HFD-fed hyperlipidemic mice. The decoy ODN technique was employed to block transcription factor activity through the use of a synthetic double-stranded ODN containing consensus sequences of DNA binding sites, which works to block mRNA transcription at the DNA level. This has been proposed as an effective therapeutic tool for inhibiting specific gene expression in several medical conditions [32,33,34]. Our findings indicate that SREBP decoy ODN reduced inflammation cytokines, alleviated hyperlipidemia and decreased hepatic lipid accumulation. As a new treatment for hyperlipidemia, synthetic SREBP decoy ODN was used to suppress the SREBP transcription factor.

SREBPs can accelerate the expression of lipid uptake and the lipid biosynthesis gene [35]. SREBPs, members of the basic helix-loop-helix leucine zipper family of transcription factors, are modulate the enzymes responsible for the synthesis of cholesterol, fatty acids, and triglycerides [13]. The SREBP-1c improves the transcription of genes needed for fatty acid synthesis [36]. SREBP-2 is principally responsible for the cholesterol-related genes, such as HMGCR, a restriction enzyme in cholesterol synthesis, and the low-density lipoprotein receptor (LDLR) gene. Both SREBP-1a and SREBP-1c are encoded by a single gene employing substitute transcription start sites that produce alternate forms of exon [13,37,38]. In spite of their obvious roles in lipid metabolism, SREBPs are all composited as endoplasmic reticulum membrane proteins and are stimulated by proteolytic cleavage in the golgi through the same processing route [39]. Due to SREBPs play a critical role in lipid synthesis, inhibition of SREBPs may be a valuable strategy for treating type II diabetes, insulin resistance, atherosclerosis, and fatty liver disease [35].

Hepatic fat deposition results from an imbalance between lipid acquisition and lipid disposal. This is modulated through four crucial pathways: uptake of circulating lipids, de novo lipogenesis, fatty acid oxidation, and the export of lipids in very low-density lipoproteins [40]. A novel lipogenesis allows the liver to synthesize new fatty acids from acetyl-CoA. Acetyl-CoA is converted to malonyl-CoA via ACC and malonyl-CoA and acetyl-CoA are then transmuted to palmitate by FAS to help form saturated fatty acids [40]. FAS is a multifunctional protein that synthesizes the saturated fatty acid palmitate from acetyl CoA, malonyl CoA, and NADPH [41]. In addition, the promoter region of FAS includes SRE [42]. In this way, FAS plays a main role in de novo fatty acid synthesis and in the long-term, control of lipogenesis [43]. SCD-1, which is a central player in lipid metabolism, is a regulatory enzyme in lipogenesis, catalyzing the rate-limiting step in the overall de novo synthesis of unsaturated fatty acids from acyl-CoA substrates [44,45]. The SCD-1 gene is regulated at the transcription level by several dietary factors such as, cholesterol, polyunsaturated fatty acids, glucose, and fructose in a SREBP-1c-dependent mechanism [45]. Various studies certify that SREBP-1c regulates the expression of numerous genes connected to fatty acid and triglyceride synthesis including SCD-1, FAS, ACC and GPAT through the insulin signaling pathway [45]. The current study aimed to understand the mechanism by which SREBP decoy ODN regulates liver lipid metabolism. The protein expression levels of the lipogenesis-related genes, SREB-1c, ACC, FAS, and SCD-1, in the liver were measured by western blot analysis. Interestingly, HFD-fed mice had a robust increase in the hepatic expression levels of lipogenic genes, including SREB-1c, ACC, FAS, and SCD-1. In contrast, SREBP decoy ODN decreased the expression of genes involved in the fatty acid synthetic pathway, such as FAS, SCD-1, ACC1. Our results show that SREBP decoy ODN decreased the SREBP-1c, FAS, ACC1, and SCD-1 protein levels in the liver. These results suggest that SREBP decoy ODN inhibits the SREBP signaling pathway that is activated by HFD. 

In addition, SREBP decoy ODN decreased AST, ALT, total cholesterol, and triglyceride levels in the serum of hyperlipidemic mice fed with HFD, and furthermore, significantly inhibited the pro-inflammatory cytokine expressions of IL-6, TNF-α, IL-1β, and IL-8 in HFD-induced hyperlipidemia mouse models. Our findings also confirm that SREBP decoy ODN mitigates damage to the arteries of HFD-induced hyperlipidemic mice. 

Our results suggest that SREBP decoy ODN exerts its anti-hyperlipidemia effects in HFD-induced hyperlipidemia mice by regulating their lipid metabolism and inhibiting lipogenesis through inactivation of the SREPB pathway. From this perspective, SREBP decoy ODN may be an effective agent for treating hyperlipidemia and hyperlipidemia-related comorbidities. 

## 4. Materials and Methods

### 4.1. Construction of Decoy ODNs

The target sites for SREBP were selected via the sequential overlap-simulation of secondary structures using the S-Fold program (Figure 1). Synthetic decoy ODNs were synthesized on a Macrogen. Synthetic ODN sequences were used as follows (the target site of consensus binding sequence is underlined): scrambled (Scr) ODN: 5′- GAATTCCAGGTACGAAGCAAAAGCTTCGTACCTG-3′; SREBP decoy ODN (consensus sequence is underlined): 5′-GAATTCGTGTGGGGTGATTGAAAACA ATCACCCCACAC-3′. Scr decoy ODN and SREBP decoy ODN were annealed for 6 h, while the temperature was decreased from 80 °C to 25 °C To obtain a covalent ligation for ring-type decoy ODN, each ODN was mixed with T4 ligase (Takara Bio Inc., Kusatsu, Japan) and incubated for 18 h at 16 °C.

### 4.2. Animal Model

Six-week-old male C57BL/6 mice (Samtako Inc., Osan, Korea) were individually housed in polycarbonate animal cages and maintained under a constant temperature (22 ± 2 °C) and humidity (55%). All animal protocols were approved by the Institutional Animal Care and Use Committee of Catholic University of Daegu (Daegu, Korea; DCIAFCR-151007-8-Y (07 October 2015)). The mice had free access to water and food and were subjected to 12 h light-dark cycles. The mice were randomly divided into four groups (6 mice/group) as follows: (1) an untreated group (normal control, NC); (2) a SREBP decoy ODN treated group (SREBP ODN); (3) HFD fed with scrambled decoy ODN treated group (HFD + Scr ODN); (4) HFD fed with SREBP decoy ODN treated ODN (HFD + SREBP ODN). The HFD group mice were fed with a high-fat diet containing of 21% fat, 2% cholesterol, 24% protein, and 41% carbohydrate for 12 weeks (Hyochang science, Daegu, Korea). The SREBP decoy ODN or scrambled decoy ODN (10 μg) was transferred every two weeks for 12 weeks via the mouse tail vein injection, using a Trans IT in vivo gene delivery system (Mirus Bio, WI, USA). All animals were anesthetized with isoflurane inhalation (Ifran; HANA Pharm, Seoul, Korea) by using RC2 Rodent Circuit Controller (VETEQUIP, CA, USA). The mice were sacrificed 12 weeks after fed HFD. At the end of each treatment period, blood samples were collected by cardiac puncture from the mice, and the mice were sacrificed by CO_2_ asphyxiation.

### 4.3. Histological Analysis

All liver and aorta tissue specimens were fixed in 10% formalin for 24 h at RT. After fixation, perpendicular sections to the anterior–posterior axis of the tissue were dehydrated in graded ethanol, cleared in xylene, and embedded in paraffin. The sections (4 μm) were mounted on glass slides, rehydrated in distilled water, and stained with hematoxylin and eosin (H&E). As part of the histological assessment, all slides were examined under a slide scanner (3DHISTECH Pannoramic MIDI, Budapest, Hungary).

### 4.4. Immunohistochemical Staining

Paraffin-embedded tissue sections of 4 μm thicknesses were deparaffinized with xylene, dehydrated in gradually diminishing concentrations of ethanol, and treated with 3% hydrogen peroxidase in methanol for 10 min to block endogenous peroxidase activity. The tissue sections were immersed in a 10 mM sodium citrate buffer (pH 6.0) for 5 min at 95 °C. The last step was repeated using a 10 mM sodium citrate solution (pH 6.0). The sections were allowed to stay in the same solution while cooling for 20 min, and they were then rinsed in PBS. The sections were then incubated with a primary antibody (1:100 dilution) for 1 h at 37 °C. The primary antibody was follows: anti-HMGCR (Abcam, Cambridge, UK). The signal was visualized using an Envision System (DAKO, Carpinteria, CA, USA) for 30 min at 37 °C. 3,3′-diaminobenzidine tetrahydrochloride (DAB) was used as the coloring reagent, and hematoxylin was used as the counter-stain. The slides were examined with a slide scanner, Pannoramic MIDI, and analyzed with iSolution DT software.

### 4.5. Immunofluorescence Staining and Confocal Microscopy

The paraffin-embedded tissue sections were deparaffinized with xylene and dehydrated in gradually decreasing concentrations of ethanol. The tissue sections were then placed in a blocking serum (5% bovine serum albumin in PBS) at RT for 1 h. A primary antibody (1:500 dilution) was incubated at RT for 2 h, and a secondary antibody incubation (1:200 dilution) was performed at RT for 1 h. The antibodies included FAS (Abcam), and a goat anti-rabbit secondary antibody conjugated with Alexa Fluor 488 (Thermo Scientific, Waltham, MA, USA). Sections were then counterstained with Hoechst 33,342. The slides were mounted using a VECTASHIELD Mounting Medium (VECTOR Laboratories, Burlingame, CA, USA). The stained slides were then viewed under a confocal microscope system (Nikon A1 microscope equipped with a digital camera, Nikon, Tokyo, Japan).

### 4.6. Serum Biochemical Analysis

Serum samples were stored at −70 °C until analyzed using a QuantiChrom™ kit (BioAssay Systems, Atlanta, GA, USA) for aspartate aminotransferase (AST) and alanine transaminase (ALT), and using an EnzyChrom™ kit (BioAssay Systems) for total cholesterol, Triglyceride Colorimetric Assay Kit (Cayman Chemical, Ann Arbor, MI, USA) for triglycerides.

### 4.7. Enzyme-Linked Immunosorbent Assay (ELISA)

Mice serum levels of interleukin-6 (IL-6, R&D Systems, Minneapolis, MN, USA) were measured using-ELISA kits according to the manufacturer’s instructions. Briefly, the sample serum and standard solutions were added to plates pre-coated with specific antibodies for IL-6, and they were incubated for 1 h at RT. Horseradish peroxidase-conjugated mouse secondary detection antibodies were then added to each well and incubated for 1–2 h at RT. Substrate solution was added to each well and incubated for 20 min at RT for color development. Finally, stop solution was added to each well, and the enzyme substrate reaction was measured using a microplate ELISA reader (BMG Labtech, Ortenaukreis, Germany) at a wavelength of 450 nm. After triplicate measurements, the concentrations of IL-6 in the samples were detected by comparison with a standard curve.

### 4.8. Real-Time PCR

Total RNA was extracted from liver tissues using the TRIzol method (Invitrogen, Carlsbad, CA, USA) according to the manufacturer’s protocol. RNA was reverse transcribed using the AccuPower RT PreMix kit (Bioneer, Daejeon, Korea). Real-time PCR was performed for an initial denaturation of 10 min at 95 °C, followed by 45 cycles of amplification for 20 s at 95 °C, 20 s at 60 °C, and 20 s at 72 °C using the LightCycler Nano Instrument Real-Time PCR System (Roche Life Science, Basel, Switzerland) with a FastStart Essential DNA Green Master Kit (Roche). The cDNA was amplified by real-time PCR with the following primers: TNF-α, 5′-CTATCTCCAGGTTCTCTTCAA-3′ (forward) and 5′-GCAGAGAGGAGGTTGACTTTC-3′ (reverse), IL-1β, 5′-GCCCATCCTCTGTGACTCA-3′ (forward) and 5′-AGTGCAGCTGTCTAATGGGA-3′ (reverse), and IL-8, 5′-TCCAATTCGGGAGACCTCTA-3′ (forward) and 5′-TAGGCATCACTGCCTGTCAA-3′ (reverse).

### 4.9. Western Blot Analysis

The liver tissues were homogenized in a protein lysis buffer for 20 min on ice and centrifuged at 12,000 rpm for 20 min at 4 °C. The supernatant was collected and the protein concentration was measured by the Bradford protein assay. Sodium dodecyl sulfate polyacrylamide gel electrophoresis was carried out with 8–12% polyacrylamide gels at 100 V for 1 h. The resolved proteins were transferred from the gel onto a nitrocellulose membrane (Millipore, Billerica, MA, USA) and probed with anti-SREBP-1c, anti-FAS, and anti-CHREBP (Abcam, Cambridge, MA, USA), anti-ACC and anti-SCD-1 (Santa Cruz Biotechnology, Santa Cruz, CA, USA), cleaved caspase-3 and anti-glyceraldehyde-3-phosphate dehydrogenase (GAPDH) (Cell Signaling Technology, Beverly, MA, USA). This was followed by a secondary antibody conjugated to horseradish peroxidase (1:1000) and determined with enhanced chemiluminescence reagents (Amersham Biosciences, Piscataway, NJ, USA). The signal intensity was quantified by an image analyzer (Chemidoc XRS+ system; Bio-Rad Laboratories, Hercules, CA, USA).

### 4.10. Electrophoretic Mobility Shift Assay (EMSA)

Nuclear extract fractionation from mice liver tissue was performed using an NE-PER Nuclear and Cytoplasmic Extraction Kit (Thermo Fisher Scientific, Waltham, MA, USA) according to the manufacturer’s instructions. Lightshift Chemiluminescent EMSA Kit (Thermo Fisher Scientific) was employ for the EMSA to analyze the expression of SREBP. ODNs composed the consensus SREBP-binding site (forward 5′-AGTCATCACCCCACTA-3′, reverse 5′-TAGTGGGGTGATGACT-3′) was used as primer.

### 4.11. Statistical Analysis

All data are presented as means ± standard error of the mean (SEM). Statistical significance was tested by one-way analysis of variance with Tukey’s multiple comparison test. Differences with *p* < 0.05 were considered significant.

## Abbreviations

NAFLD	Non-alcoholic fatty liver disease
SREBP	Sterol regulatory element binding protein
ODN	Oligodeoxynucleotide
HFD	High-fat diet
SRE-1	Sterol regulated element-1
ALT	Alanine aminotransferase
AST	Aspartate aminotransferase
FAS	Fatty acid synthase
ACC	Acetyl-CoA carboxylase
SCD	Stearoyl-CoA desaturase
HMGCR	HMG-CoA reductase
ChREBP	Carbohydrate response element binding protein
